# An ultra-wide rejection suppressing cell for microwave applications

**DOI:** 10.1371/journal.pone.0348430

**Published:** 2026-07-06

**Authors:** Farzin Shama, Milad Ekhteraei, Mohsen Hayati, Hamed Abbasi

**Affiliations:** 1 Department of Electrical Engineering, Ker.C., Islamic Azad University, Kermanshah, Iran; 2 Department of Biomedical Engineering, School of Medicine, Kermanshah University of Medical Sciences (KUMS), Kermanshah, Iran; 3 Electrical Engineering Department, Faculty of Engineering, Razi University, Tagh-E-Bostan, Kermanshah, Iran; Zhejiang University, CHINA

## Abstract

This paper presents the design and fabrication of a novel suppressing cell (SC) implemented on a RO4003 substrate for microwave circuit applications. Experimental results indicate that the SC functions as a low-pass filter (LPF) with a 3 dB cutoff frequency at 5.7 GHz and a transition band of 0.6 GHz from −3 dB to −20 dB. The design achieves an ultra-wide stopband from 6.3 GHz to 50 GHz, with significant attenuation noted in the S22 parameter around 38 GHz, effectively covering five harmonics of the cutoff frequency. The return loss in the passband is measured at 10 dB, with an insertion loss of just 0.5 dB at 1.3 GHz. Additionally, the size of the SC is compact at 15.3 mm × 10.5 mm (15.3 λg × 10.5 λg), and the group delay in the passband exhibits minimal variation, recorded at only 0.53 ns.

## Introduction

The rapid advancement of microwave technology has necessitated the development of innovative filtering solutions capable of addressing the increasing demands for signal integrity and performance in communication systems. As applications in wireless communications, radar, and satellite systems expand, along with the need for a specified bandwidth selection [1–19] the need for ultra-wideband (UWB) rejection filters has become paramount [2]. Traditional filtering techniques often struggle to provide sufficient selectivity and bandwidth, leading to the emergence of novel designs that can effectively suppress unwanted signals while maintaining desired frequency characteristics. In this context, the design of an ultra-wide rejection suppressing cell presents a promising solution for enhancing microwave systems’ performance. Such cells are engineered to achieve significant attenuation across a broad range of frequencies, thereby mitigating interference from out-of-band signals and improving overall system reliability. Recent advancements in materials science and circuit design have facilitated the development of compact and efficient filter structures that exhibit superior rejection capabilities without compromising insertion loss or bandwidth. Today, the use of planar microstrip structures is the least costly and simplest way to design various types of filters and suppressing cells [3]. The microstrip lowpass-type suppressing cells have been widely used in many microwave circuits such as power amplifiers [4–7], power dividers [8–10], diplexers [11–12] and couplers [13–14]. In [15], a microstrip lowpass suppressing cell has been integrated with a class-F power amplifier to eliminate unwanted harmonics. A simple stepped impedance structure and compact size are the benefits of this cell but the frequency response of this lowpass filter is not appropriate at all. In [16], employing tapered step impedance resonators, an ultra-wide stopband LPF has been designed with a non-sharp transition band. In [17], the defected ground structure (DGS) technique technique has been used to fabricate a wide stopband LPF, which is a mostly high-cost and complex method in comparison to the planar structures. In [20], a compact microstrip low-pass filter is presented featuring a sharp transition response achieved through the use of coupled radial stubs. The design maintains very low loss within the passband while employing bent transmission lines with stepped-impedance cells to effectively extend the stopband over a wide frequency range. However, the design complexity introduced by the combination of radial stubs and stepped-impedance sections may increase fabrication sensitivity and tolerance issues. A 1 GHz microstrip low-pass filter is designed in [21] based on modified Jacobi polynomials, where transmission zeros are introduced to improve selectivity and passband performance. Among the evaluated seventh-order designs, the configuration with simple transmission zeros shows a sharp roll-off; however, the return loss value in the passband is questionable, and it also has a narrow stopband width. A compact microstrip low-pass filter with sharp transition characteristics and a wide stopband is presented in [22], achieved through the use of a modified resonator and additional stub elements. High selectivity and strong suppression performance are obtained; however, increased structural complexity and relatively large size may be considered as potential drawbacks for compact integrations. In [23], a low-profile microstrip low-pass filter is proposed using a T-shaped structure and a defected ground structure to achieve sharp roll-off and wide stopband performance. While the design offers good selectivity and harmonic suppression, it may suffer from increased three-dimensional (3D) fabrication complexity and large size. A compact microstrip low-pass filter using meandered-line resonators and radial stubs is proposed in [24], offering sharp roll-off and a wide stopband. While it provides high selectivity and small size, it may involve increased design complexity and sensitivity to fabrication tolerances with a low return loss in the passband region.

This paper presents a comprehensive analysis of an ultra-wide rejection suppressing cell tailored for microwave applications. The findings are expected to contribute significantly to the ongoing research in microwave engineering and offer valuable insights for future developments in filtering technology.

## Suppressing cell design

**Fig 1:**
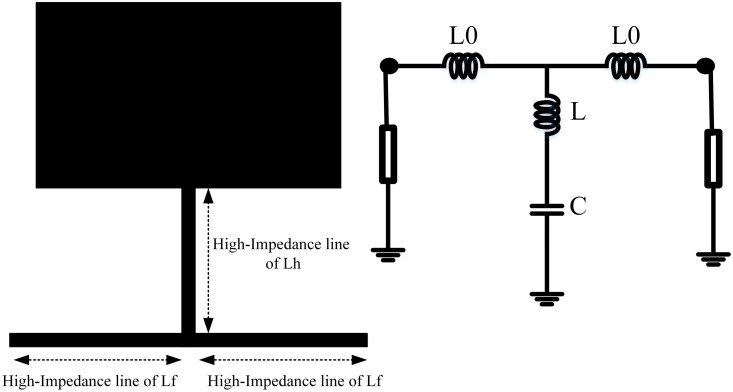
A conventional T-shaped resonator.


A T-shaped resonator is conventionally used to have an elliptical frequency response, which has a sharp transition band. It contains a square-shaped patch connected to a high-impedance line of *Lh* and fed by other high-impedance lines of *Lf*s as illustrated in Fig 1 with its *LC* model, where *L*_*0*_ represents the high-impedance lines of *Lf*s; *L* represents the high-impedance line of *Lh,* and *C* represents the low-impedance patch*.* By considering an ohmic load of *R*_*L*_ connected to the output port, by applying KCL to this circuit:

$$\begin{array}{c} \frac{{V_{i-}V}_{\text{0}}}{{j\omega L}_{\text{0}}}+\frac{{V_{i-}V}_{s}}{{j\omega L}_{\text{0}}}+V_{i}\left(\frac{{V_{i-}V}_{\text{0}}}{j\omega L+\frac{\text{1}}{j\omega C}}\right)=\text{0} \end{array}$$ (1)

$$\begin{array}{c} \frac{{V_{o-}V}_{i}}{{j\omega L}_{\text{0}}}+\frac{V_{o}}{R_{L}}=\text{0} \end{array}$$ (2)

By Combining Equations (1) and (2), and simplifying the equations a transfer function can be defined as below:


$$\begin{array}{c} \frac{V_{\text{0}}}{V_{s}}=\frac{\frac{R_{L}}{R_{L}+{j\omega L}_{\text{0}}}}{\left(\frac{{-\omega }^{\text{2}}L_{\text{0}}C}{\text{1}-\omega^{\text{2}}LC}\right)-\frac{R_{L}}{R_{L}+{j\omega L}_{\text{0}}}+\text{2}} \end{array}$$ (3)

**Fig 2:**
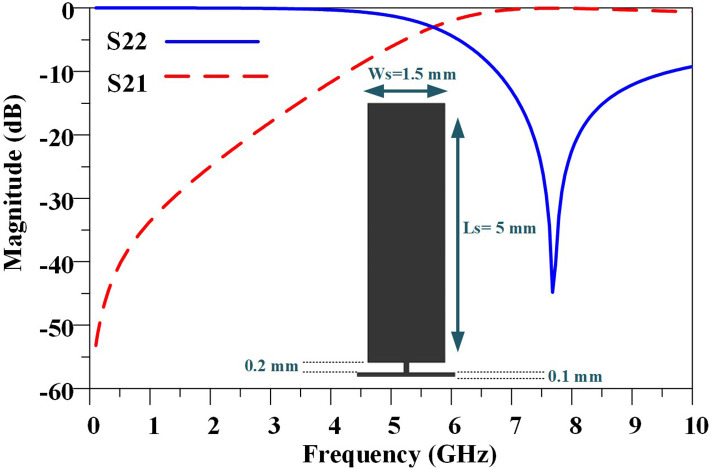
Rectangular-shaped resonator.

But to design a suppressing cell with an ultra-wide rejection band for high frequencies the sharpness of the transition band is of second-order importance. So, to have a wider rejection band and move a transmission zero to a higher frequency, the T-shaped resonator will be changed to a rectangular-shaped resonator with a short high-impedance line of *L*_*h*_ as shown in Fig 2. It has been shown with its simulated S-parameters. As illustrated, it has a transmission zero at 7.68 GHz with an attenuation level of 44.8 dB. Also, by considering the ideal attenuation level of 20 dB, it provides a 0.7 GHz rejection band, which is very narrow.

**Fig 3:**
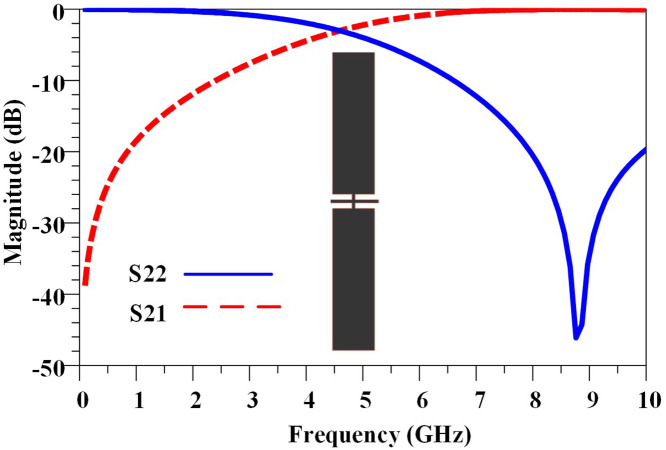
Mirrored rectangular-shaped resonator.

Equation 3 shows that to move the transmission zero to a higher frequency, and then have a wider rejection region the value of *C* must be increased while the value of *L* must be decreased. By the symmetric rules for lumped-element circuits, it is known that a mirrored symmetric structure doubles the capacitors and halves the inductors. So, the rectangular-shaped resonator will be mirrored as shown in Fig 3. As illustrated in this figure, the transmission zero moved to 9.44 GHz with an attenuation level of 51 dB. Also, by considering the ideal attenuation level of 20 dB, it provides a 2 GHz rejection band, which is still narrow.

**Fig 4:**
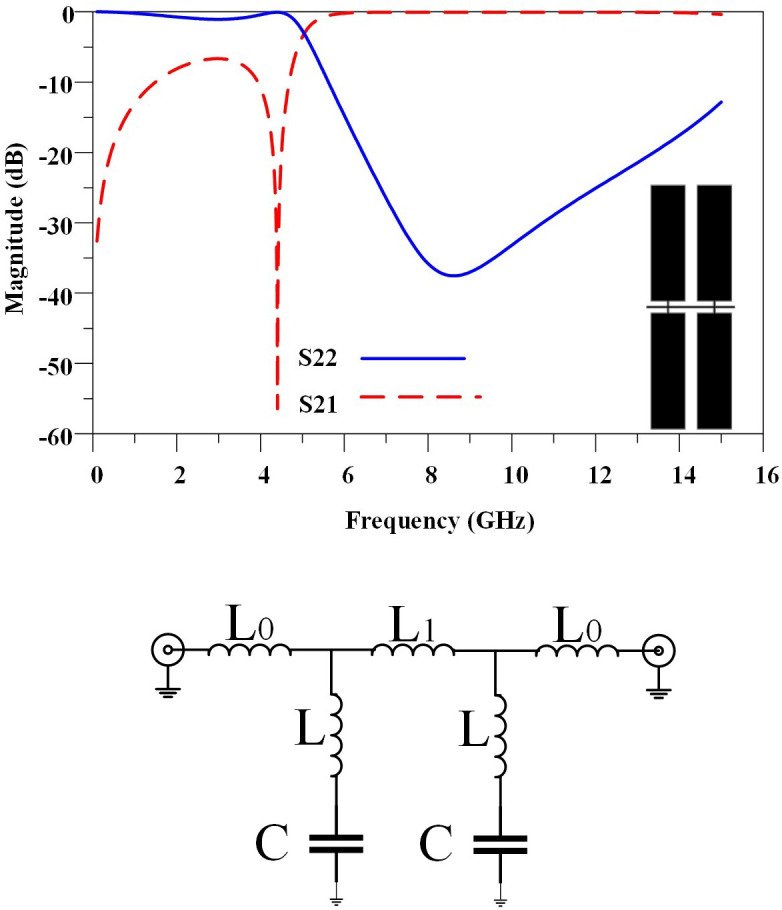
Cascaded mirrored rectangular-shaped resonator.

**Fig 5:**
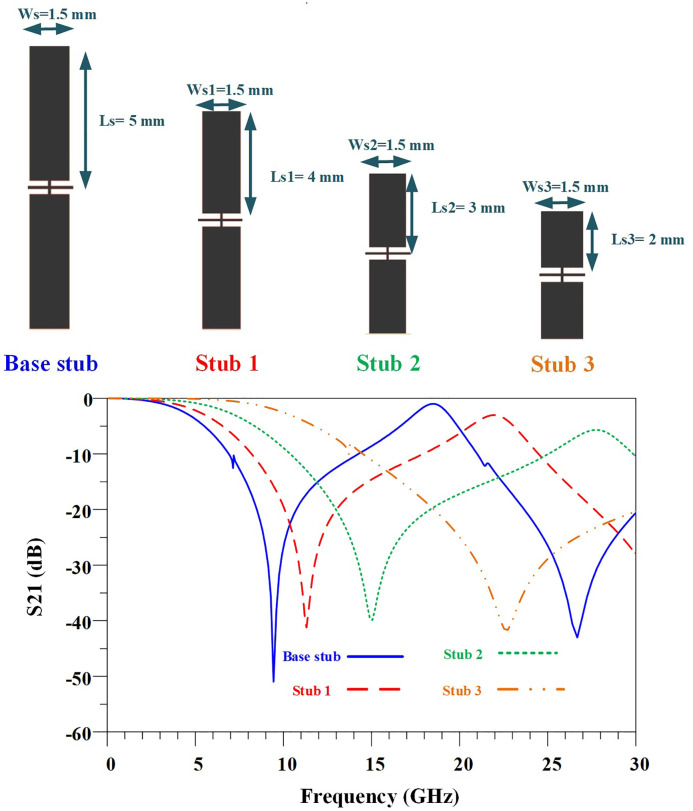
New designed resonators with various stub sizes.

Another method to create a wider rejection region is cascading two similar resonators together by taking advantage of adding two transmission zeros with high level attenuation. This method will be applied as shown in Fig 4. As illustrated in this figure, by considering the ideal attenuation level of 20 dB, it provides a 7 GHz rejection band, from 6.4 GHz to 13.4 GHz. It is all the result of using these rectangular-shaped resonators with these dimensions and related transmission zeroes. Using Equation (3), it is clear that by decreasing the value of capacitor *C*, the transmission zero can be moved to a higher frequency. So. Three resonators will be designed with various stub sizes as illustrated in Fig 5. As illustrated in this figure, stub 1 creates a transmission zero at 11.3 GHz with an attenuation level of 41 dB. Stub 2 creates a transmission zero to 15 GHz by attenuation level of 40 dB; and stub 3 creates a transmission zero at 21.7 GHz with an attenuation level of 41.5 dB. The aim is to add all of these new resonators with these new stubs to the cascaded mirrored rectangular-shaped resonator with a base stube in a similar form. To have a better perspective for showing the effect of adding new transmission zeros to the frequency response by each used stub, they have been added in a step-by-step procedure.

**Fig 6:**
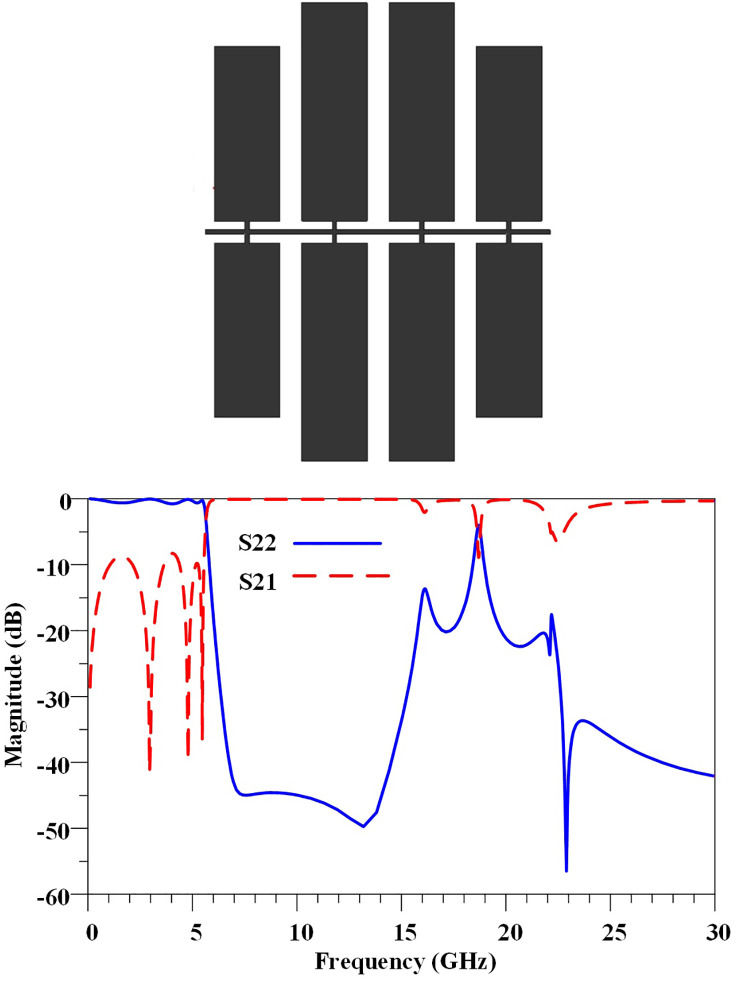
Cascaded mirrored rectangular-shaped resonator plus stub 1.

At the first step the resonator with stub 1 will be added as shown in Fig 6. As illustrated in this figure, by adding a new transmission zero, and by considering the ideal attenuation level of 20 dB, it provides a 9.7 GHz rejection band, from 6 GHz to 15.7 GHz. Clearly, the transition band has been sharper using this method.

**Fig 7:**
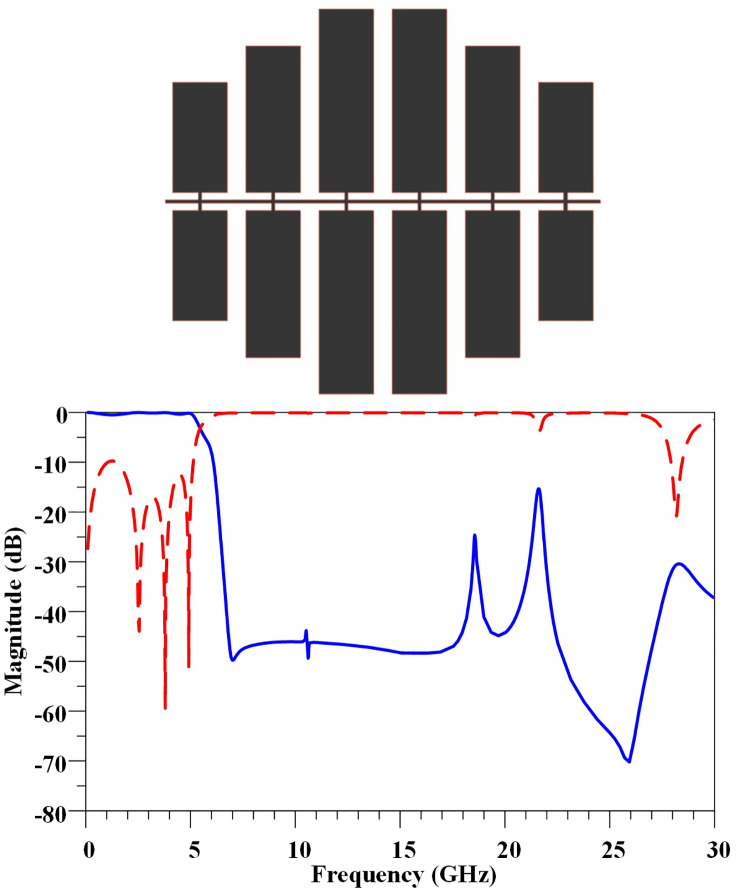
Cascaded mirrored rectangular-shaped resonator plus stub 1 and stub 2.

At the second step the resonator with stub 2 will be added as shown in Fig 7. As illustrated in this figure, by adding a new transmission zero, and by considering the ideal attenuation level of 20 dB, it provides a significantly increased 15.1 GHz rejection band, from 6.3 GHz to 21.4 GHz (Fig 7).

**Fig 8:**
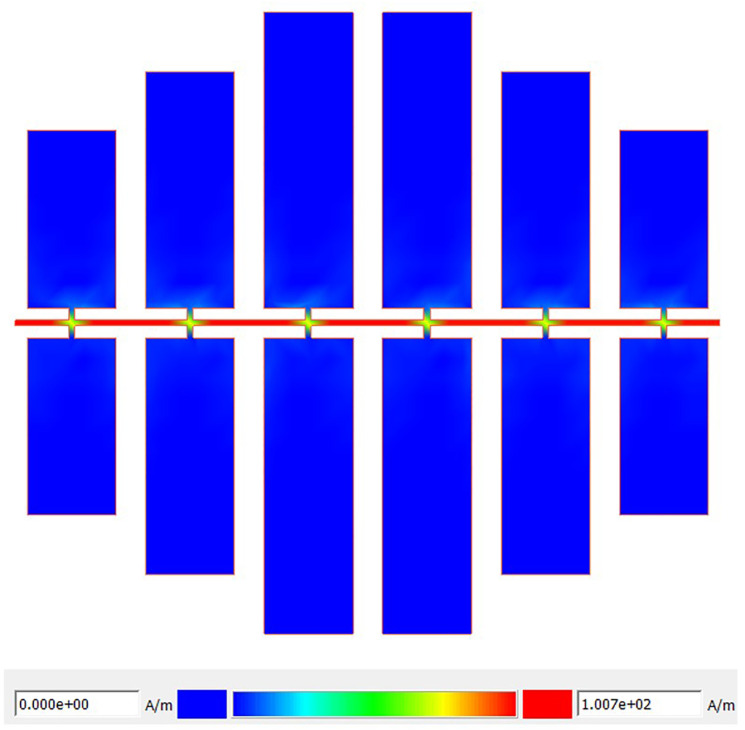
Simulated current density of Cascaded mirrored rectangular-shaped resonator plus stub 1 and stub 2.

**Fig 9:**
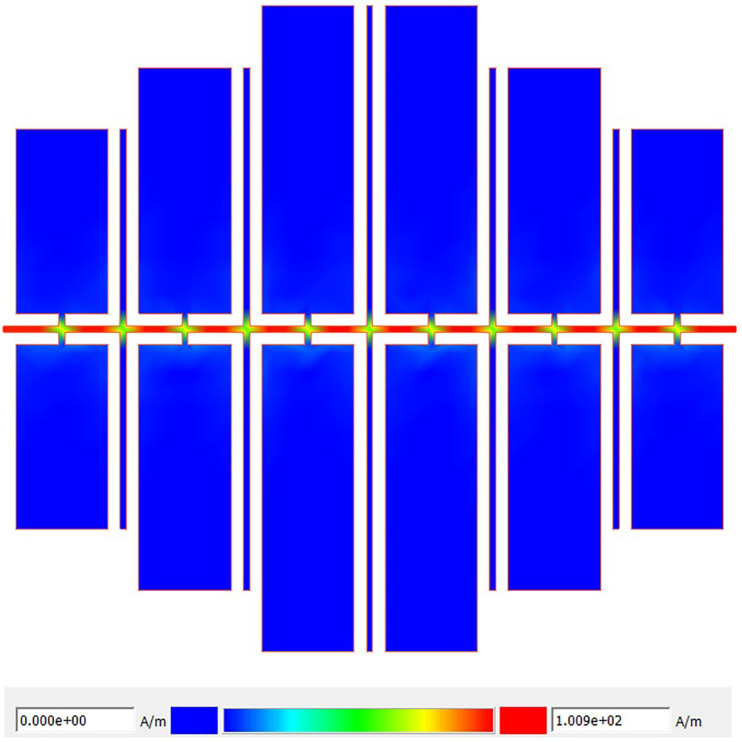
Simulated current density of modified suppressing cell.

Before adding the final resonator with stub 3, another method is to use the extra space between the resonators in Fig 8, a modified suppressing cell can be designed as illustrated in Fig 9, with its simulated current density. As a result, the S-parameters do not change at all but the maximum current density in the feeding line is increased while there is no physical size increment.

**Fig 10:**
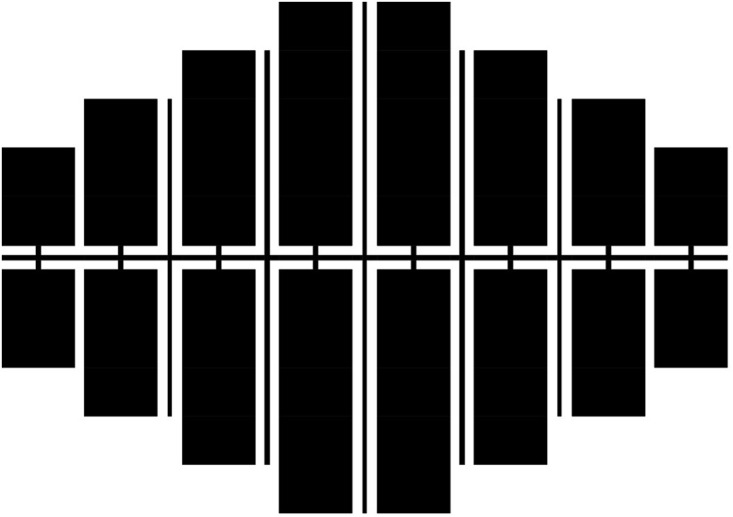
Final structure of the proposed suppressing cell.

**Fig 11:**
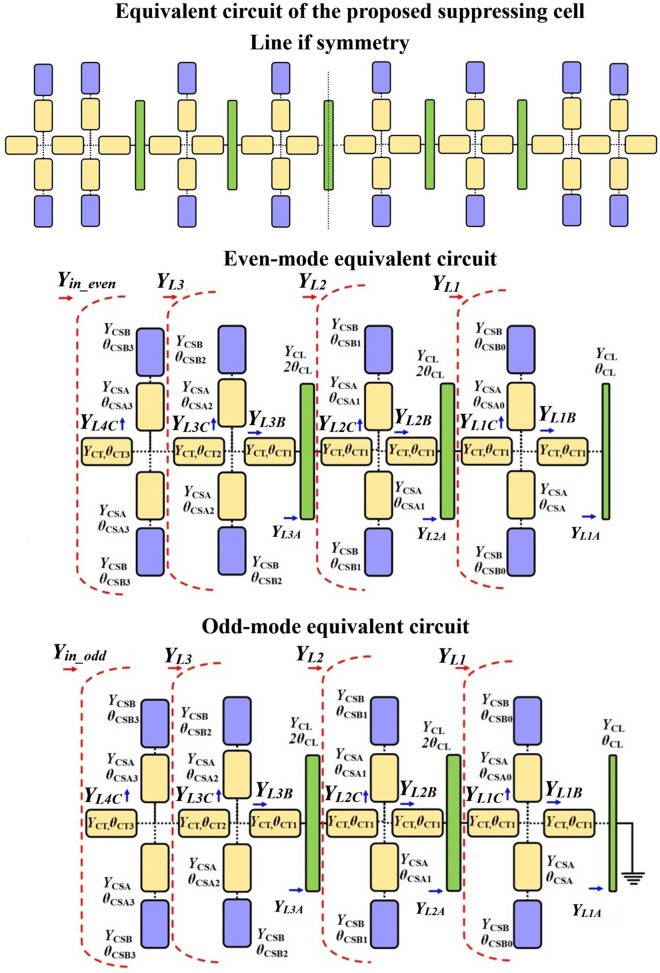
The equivalent circuit of the proposed suppressing cell along with its even- and odd-mode equivalent circuits.

At the final step the resonator with stub 3 will be added to the modified suppressing cell as shown in Fig 10 to create an ultra-wide stopband suppressing cell. As illustrated in this figure, by adding a new transmission zero, and by considering the ideal attenuation level of 20 dB, it provides an incredibly increased rejection band, as shown in Fig 11.

In Fig 11, the equivalent circuit of the proposed suppressing cell along with its even- and odd-mode equivalent circuits are illustrated based on the characteristic admittance *Y* and electrical length *θ* of each section of the structure. Cells with the same color have equal characteristic impedances. Since the proposed structure is symmetrical, even- and odd-mode analysis will be presented for this structure.

The even-mode analysis is presented based on the equivalent circuit shown in Fig 11. As can be observed from the structure, the circuit consists of repeating sections with similar configurations, which simplifies the analysis. For the first section of the circuit, whose admittance is denoted by *Y*_L1_, we have:


$$\begin{array}{c} Y_{L\text{1}A\_even}=-jY_{CL}\text{cot}\left(\theta_{CL\text{1}}\right) \end{array}$$ (4)

$$\begin{array}{c} Y_{L\text{1}B\_even}=Y_{CT}\frac{Y_{L\text{1}A\_even}+jY_{CT}\text{tan}(\theta_{CT\text{1}})}{Y_{CT}+jY_{L\text{1}A\_even}\text{tan}(\theta_{CT\text{1}})} \end{array}$$ (5)

$$\begin{array}{c} Y_{L\text{1}C}=Y_{CSA}\frac{-jY_{CSB}\text{cot}\left(\theta_{CSB\text{0}}\right)+jY_{CSA}\text{tan}(\theta_{CSA\text{0}})}{Y_{CSA}+Y_{CSB}\text{cot}\left(\theta_{CSB\text{0}}\right)\text{tan}(\theta_{CSA\text{0}})} \end{array}$$ (6)

$$\begin{array}{c} Y_{L\text{1}\_even}=Y_{CT}\frac{\left({\text{2}Y}_{L\text{1}C}+Y_{L\text{1}B\_even}\right)+jY_{CT}\text{tan}(\theta_{CT\text{1}})}{Y_{CT}+j\left({\text{2}Y}_{L\text{1}C}+Y_{L\text{1}B\_even}\right)\text{tan}(\theta_{CT\text{1}})} \end{array}$$ (7)

Following a similar procedure to that of *Y*_L1_, the relations for the subsequent sections, denoted by *Y*_L2_ and *Y*_L3_, are given by:


$$\begin{array}{c} Y_{L\text{2}\_even}=Y_{CT}\frac{\left({\text{2}Y}_{L\text{2}C}+Y_{L\text{2}B\_even}\right)+jY_{CT}\text{tan}(\theta_{CT\text{1}})}{Y_{CT}+j\left({\text{2}Y}_{L\text{2}C}+Y_{L\text{2}B\_even}\right)\text{tan}(\theta_{CT\text{1}})} \end{array}$$ (8)

$$\begin{array}{c} Y_{L\text{3}\_even}=Y_{CT}\frac{\left({\text{2}Y}_{L\text{3}C}+Y_{L\text{3}B\_even}\right)+jY_{CT}\text{tan}(\theta_{CT\text{2}})}{Y_{CT}+j\left({\text{2}Y}_{L\text{3}C}+Y_{L\text{3}B\_even}\right)\text{tan}(\theta_{CT\text{2}})} \end{array}$$ (9)

For the input admittance of the even-mode equivalent circuit, denoted by *Y*_in_even_ the following expression is derived:


$$\begin{array}{c} Y_{in\_even}=Y_{CT}\frac{\left({\text{2}Y}_{L\text{4}C}+Y_{L\text{3}\_even}\right)+jY_{CT}\text{tan}(\theta_{CT\text{3}})}{Y_{CT}+j\left({\text{2}Y}_{L\text{4}C}+Y_{L\text{3}\_even}\right)\text{tan}(\theta_{CT\text{3}})} \end{array}$$ (10)

For the odd-mode analysis based on the equivalent circuit shown in Fig 11, a procedure similar to that of the even-mode analysis is followed. However, since there is a short circuit at the line of symmetry, the expression for *Y*_L1A_ becomes:


$$\begin{array}{c} Y_{L\text{1}A\_odd}=jY_{CL}tan\left(\theta_{CL\text{1}}\right) \end{array}$$ (11)

As previously stated, similar to the even-mode analysis, the expression for *Y*_in_odd_ is given by:


$$\begin{array}{c} Y_{in\_odd}=Y_{CT}\frac{\left({\text{2}Y}_{L\text{4}C}+Y_{L\text{3}\_odd}\right)+jY_{CT}\text{tan}(\theta_{CT\text{3}})}{Y_{CT}+j\left({\text{2}Y}_{L\text{4}C}+Y_{L\text{3}\_odd}\right)\text{tan}(\theta_{CT\text{3}})} \end{array}$$ (12)

The reflection coefficients for the even and odd modes are given by:


$$\begin{array}{c} \Gamma_{even}=\frac{Y_{\text{0}}-Y_{in\_even}}{Y_{\text{0}}+Y_{in\_even}}=\frac{Y_{\text{0}}-Y_{CT}\frac{\left({\text{2}Y}_{L\text{4}C}+Y_{L\text{3}\_even}\right)+jY_{CT}\text{tan}(\theta_{CT\text{3}})}{Y_{CT}+j\left({\text{2}Y}_{L\text{4}C}+Y_{L\text{3}\_even}\right)\text{tan}(\theta_{CT\text{3}})}}{Y_{\text{0}}+Y_{CT}\frac{\left({\text{2}Y}_{L\text{4}C}+Y_{L\text{3}\_even}\right)+jY_{CT}\text{tan}(\theta_{CT\text{3}})}{Y_{CT}+j\left({\text{2}Y}_{L\text{4}C}+Y_{L\text{3}\_even}\right)\text{tan}(\theta_{CT\text{3}})}} \end{array}$$ (13)

$$\begin{array}{c} \Gamma_{odd}=\frac{Y_{\text{0}}-Y_{in\_odd}}{Y_{\text{0}}+Y_{in\_odd}}=\frac{Y_{\text{0}}-Y_{CT}\frac{\left({\text{2}Y}_{L\text{4}C}+Y_{L\text{3}\_odd}\right)+jY_{CT}\text{tan}(\theta_{CT\text{3}})}{Y_{CT}+j\left({\text{2}Y}_{L\text{4}C}+Y_{L\text{3}\_odd}\right)\text{tan}(\theta_{CT\text{3}})}}{Y_{\text{0}}+Y_{CT}\frac{\left({\text{2}Y}_{L\text{4}C}+Y_{L\text{3}\_odd}\right)+jY_{CT}\text{tan}(\theta_{CT\text{3}})}{Y_{CT}+j\left({\text{2}Y}_{L\text{4}C}+Y_{L\text{3}\_odd}\right)\text{tan}(\theta_{CT\text{3}})}} \end{array}$$ (14)

where *Y*_0_ is the reference admittance of the input and output ports, which is equal to 0.02 S. Based on the even- and odd-mode reflection coefficients, the scattering parameters S_11_ and S_21_ are obtained using the following equations:


$$\begin{array}{c} S_{\text{2}\text{1}}=\frac{\text{1}}{\text{2}}\left(\Gamma_{even}-\Gamma_{odd}\right) \end{array}$$ (15)

$$\begin{array}{c} S_{\text{1}\text{1}}=\frac{\text{1}}{\text{2}}\left(\Gamma_{even}+\Gamma_{odd}\right) \end{array}$$ (16)

To simplify the equations, the electrical lengths of the stubs as is evident from the final layout depicted in Fig 10 satisfy the following relationship:


$$\begin{array}{c} \theta_{CSA\text{0}}=\theta_{CSA\text{1}}=\theta_{CSA\text{2}}=\theta_{CSA\text{3}} \end{array}$$ (17)

$$\begin{array}{c} \theta_{CSB\text{2}}=\text{1}.\text{5}\theta_{CSB\text{3}},\;\theta_{CSB\text{1}}=\text{2}\theta_{CSB\text{3}},\;\theta_{CSB\text{0}}=\text{2}.\text{5}\theta_{CSB\text{3}}\; \end{array}$$ (18)

To obtain the transmission zeros of the frequency response, the even- and odd-mode reflection coefficients must be equal. Therefore, we have:


$$\begin{array}{c} \frac{Y_{\text{0}}-Y_{CT}\frac{\left({\text{2}Y}_{L\text{4}C}+Y_{L\text{3}\_even}\right)+jY_{CT}\text{tan}(\theta_{CT\text{3}})}{Y_{CT}+j\left({\text{2}Y}_{L\text{4}C}+Y_{L\text{3}\_even}\right)\text{tan}(\theta_{CT\text{3}})}}{Y_{\text{0}}+Y_{CT}\frac{\left({\text{2}Y}_{L\text{4}C}+Y_{L\text{3}\_even}\right)+jY_{CT}\text{tan}(\theta_{CT\text{3}})}{Y_{CT}+j\left({\text{2}Y}_{L\text{4}C}+Y_{L\text{3}\_even}\right)\text{tan}(\theta_{CT\text{3}})}}=\frac{Y_{\text{0}}-Y_{CT}\frac{\left({\text{2}Y}_{L\text{4}C}+Y_{L\text{3}\_odd}\right)+jY_{CT}\text{tan}(\theta_{CT\text{3}})}{Y_{CT}+j\left({\text{2}Y}_{L\text{4}C}+Y_{L\text{3}\_odd}\right)\text{tan}(\theta_{CT\text{3}})}}{Y_{\text{0}}+Y_{CT}\frac{\left({\text{2}Y}_{L\text{4}C}+Y_{L\text{3}\_odd}\right)+jY_{CT}\text{tan}(\theta_{CT\text{3}})}{Y_{CT}+j\left({\text{2}Y}_{L\text{4}C}+Y_{L\text{3}\_odd}\right)\text{tan}(\theta_{CT\text{3}})}} \end{array}$$ (19)

**Fig 12:**
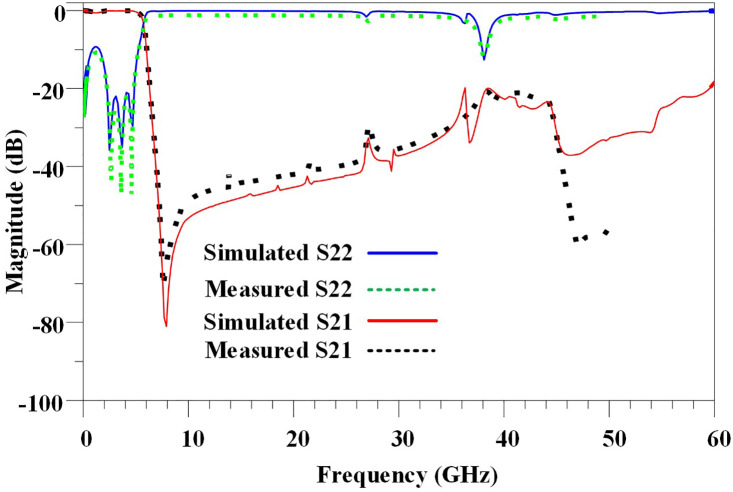
S-parameters of the proposed SC.

Therefore, to achieve a wide stopband, the dimensions have been chosen such that the transmission zeros are distributed throughout the stopband. This theoretical condition is highly consistent with the simulated scattering parameters. As will be shown in the final frequency response (Fig 12), multiple transmission zeros are strategically generated across the stopband, which validates the mathematical even- and odd-mode derivations and demonstrates the effectiveness of the proposed design in achieving a wide rejection band. By analyzing this theoretical condition, the physical dimensions of the stubs are precisely synthesized rather than relying solely on empirical tuning.

## Experimental results

**Fig 13:**
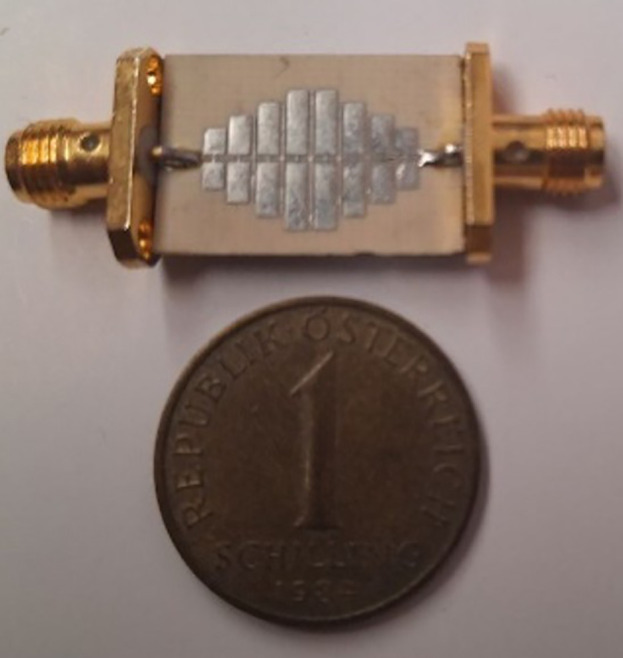
Circuit photograph.

**Fig 14:**
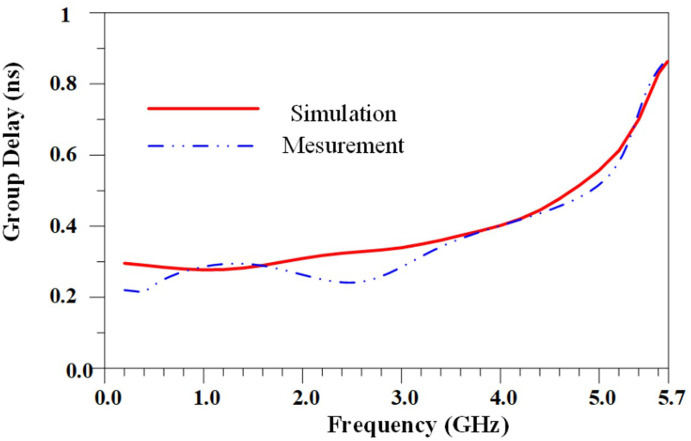
Group delay.

The designed suppressing cell (SC) has been fabricated on a suitable RO4003 substrate for microstrip circuits with a thickness of 0.508 mm, a permittivity (ε_r_) constant of 3.38, and loss tangent of 0.0022. The S-parameters and fabricated circuit have been shown in Figs 12 and 13, respectively. The Keysight N5245B network analyzer has been used for measurements, which can measure the S-parameters up to 50 GHz, while the simulated results have been shown up to 60 GHz. Based on measurements, the proposed SC acts as LPF with a 3 dB cutoff frequency at 5.7 GHz, and a transition band of 0.6 GHz from −3 dB to the −20 dB point. By considering the ideal attenuation level of 20 dB, it provides an ultra-wide stop band from 6.3 GHz to 50 GHz, while because of a significant drop in the S22 parameter at about 38 GHz, the stopband region has been considered to be 31.7 GHz, which covers at least five harmonics of the measured cutoff frequency. The maximum return loss in the passband region is 10 dB with a corresponding insertion loss of 0.5 dB at 1.3 GHz. The size of the SC is 15.3 mm × 10.5 mm, or 0.47 $\lambda_{g}$ × 0.32 $\lambda_{g}.$ In order to further strengthen the experimental validation and to increase confidence in the measured results, an additional comparative analysis has been performed, as shown in Fig 14 between the measured and simulated group delay by considering the insertion loss (S21 parameter). Fig 14 shows the calculated and measured group delay in the passband region. As observed, the maximum variation of the group delay is only 0.53 ns. In many previous researches many factors have been defined for an LPF, such as suppression factor (SF), related stopband bandwidth (RSB) (all defined in [3]), and GD, which is the maximum variation of the group delay in *ns*.

Table 1 shows a comparison between some published SCs. The comparison in Table 1 indicates that the fabricated structure (SC) operates at a higher cutoff frequency (5.7 GHz) while maintaining competitive performance in terms of selectivity factor (SF = 2) and relative stopband bandwidth (RSB = 1.48), comparable to or better than several reported works such as [2,6], and [19]. Although its group delay (0.53 ns) is slightly higher than most references, it remains within an acceptable range for practical applications. A notable advantage of the proposed design is its very simple manufacturability, in contrast to many prior works that involve relatively complex structures. However, this simplicity comes at the expense of a larger normalized circuit size compared to other designs, such as [6] and [19], which offer more compact implementations. Overall, the fabricated filter achieves a balanced trade-off between performance and ease of fabrication.

**Table 1:** Performance review.

References	f_c_ (GHz)	RSB	SF	GD (ns)	Normalized circuit size	Manufacturability
[2]	3.8	1.34	2	0.3	0.19$\lambda_{g}\times$ 0.10$\lambda_{g}$	Fairly complex
[6]	1.53	1.79	2	0.2	0.06$\lambda_{g}$ ×0.14$\lambda_{g}$	Fairly complex
[7]	4.2	1.30	2	–	0.32$\lambda_{g}$ $\times \text{0}.\text{15}\lambda_{g}$	Fairly complex
[9]	2.2	1.13	1.8	–	0.29$\lambda_{g}$ × 0.14$\lambda_{g}$	Fairly simple
[15]	4	1.39	1.6	–	0.$\lambda_{g}$19 × 0.09$\lambda_{g}$	Simple
[18]	4	1.39	1.6	0.23	0.19$\lambda_{g}$ × 0.09$\lambda_{g}$	Simple
[19]	3.5	1.62	2	0.05	0.15 $\lambda_{g}$ × 0.09 $\lambda_{g}$	Fairly complex
[25]	2.2	1.62	2	–	0.19$\lambda_{g}\times$0.08$\lambda_{g}$	Fairly simple
[26]	1.53	1.7	2	0.05	0.13$\lambda_{g}\times \text{0}.\text{06}\lambda_{g}$	Fairly complex
Fabricated SC	5.7	1.48	2	0.53	0.47 $\lambda_{g}$ × 0.32 $\lambda_{g}$	Very simple

## Conclusion

A simple, planar, ultra-wide rejection suppressing cell was designed and fabricated. The fabricated suppressing cell (SC) demonstrates significant advancements in low-pass filter design for microstrip applications, achieving a balance between compact size and superior performance characteristics. The measured parameters confirm that the SC effectively operates as an LPF with an impressive cutoff frequency and extensive stopband capabilities, thus making it a valuable component for high-frequency circuit designs. The minimal group delay variation further enhances its applicability in scenarios where signal integrity is paramount. Future work may explore optimizing the SC design for even broader bandwidths and reduced insertion losses, potentially expanding its utility across various RF and microwave applications.
